# Artificial Antigen Presenting Cells: An Off the Shelf Approach for Generation of Desirable T-Cell Populations for Broad Application of Adoptive Immunotherapy

**Published:** 2015-10-05

**Authors:** AN Hasan, A Selvakumar, RJ O’Reilly

**Affiliations:** 1 Department of Pediatrics, Memorial Sloan-Kettering Cancer Center, USA; 2 Bone Marrow Transplantation Service, Division of Bone Marrow Transplantation, Memorial Sloan-Kettering Cancer Center, USA; 3 lmmunology Program, Sloan-Kettering Institute at Memorial Sloan-Kettering Cancer Center 1275 York Avenue, New York, NY 10021, USA

**Keywords:** Immunotherapy, Antigen, Tumor

## Abstract

Adoptive transfer of antigen specific T-cells can lead to eradication of cancer and viral infections. The broad application of this approach has further been hampered by the limited availability of adequate numbers of T-cells for treatment in a timely manner. This has led to efforts for the development of efficient methods to generate large numbers of T-cells with specificity for tumor or viral antigens that can be harnessed for use in cancer therapy. Recent studies have demonstrated that during encounter with tumor antigen, the signals delivered to T-cells by professional antigen-presenting cells can affect T-cell programming and their subsequent therapeutic efficacy. This has stimulated efforts to develop artificial antigen-presenting cells that allow optimal control over the signals provided to T-cells. In this review, we will discuss the cellular artificial antigen-presenting cell systems and their use in T-cell adoptive immunotherapy for cancer and infections.

## Adoptive T-cell Therapy

**Figure 1:**
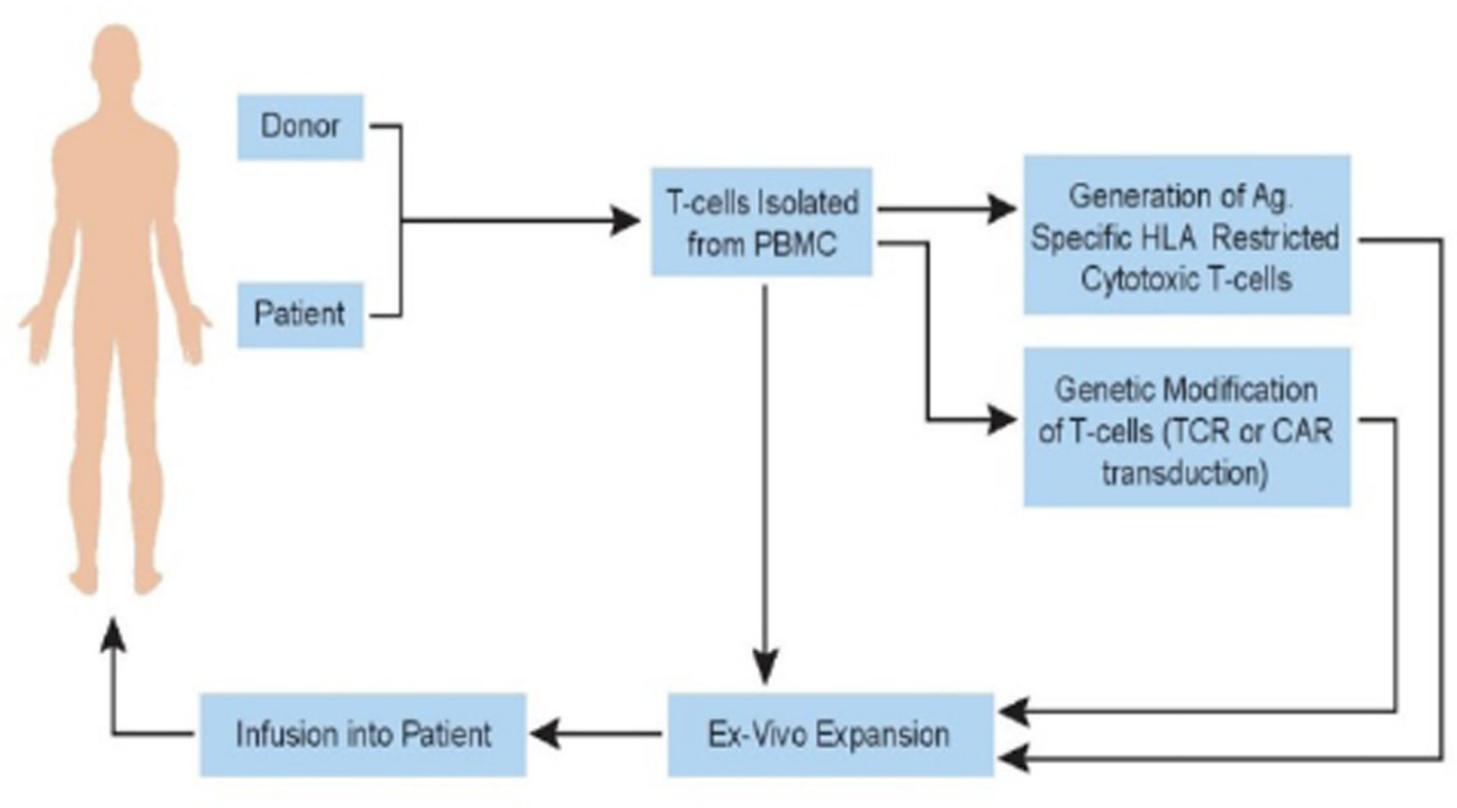
Adoptive Immunotherapy – Schematic Representation. This therapy involves the passive transfer of cellular immunity by infusion of T-cells. The T-cells for infusion can be derived from a healthy volunteer donor or from the patient themselves. In either case, the T-cells are isolated either from peripheral blood or from surgically removed tumor specimens containing infiltrating lymphocytes (TILs), and then expanded *in-vitro* to enrich for T-cells directed against specific antigens; viral or tumor antigens. In certain protocols, T-cells isolated from peripheral blood can be genetically modified to express chimeric antigen receptors which redirect the T-cells to target specific antigens expressed on tumor cells.

Targeted eradication of cancers and viral infections can be achieved with adoptive immunotherapy involving the infusion of T-cells directed against viral or tumor antigens (Figure 1). Recent clinical trials have shown that adoptive transfer of transplant donor derived virus specific T-cells have the capacity to provide protection against, and successfully eradicate EBV and CMV infections developing in recipients of hematopoietic stem cell transplants [1–5]. More recently, third party donor derived virus specific T-cells have also demonstrated effective eradication of CMV, EBV and adenoviral infections in recipients of hematopoietic stem cell transplants [6–8] and EBV infections in solid organ transplants [6].

In the treatment of cancers, immunotherapy confers higher tumor specific targeting than that afforded by conventional chemotherapy, while avoiding the off-target toxicities. Both passive and active immunity have been invoked to target and kill cancer cells. Passive immunotherapy using monoclonal antibodies targeted to specific cancer antigen overexpressed on tumor cells has demonstrated beneficial effects in several malignancies. The classic examples include anti-CD20 for lymphomas [9], and anti her-2 for breast cancer among others [10]. Similarly, transmission of active immunity by adoptive transfer of T-cells directed against specific antigens differentially expressed by tumor cells (tumor associated antigens-TAA), has emerged as an extremely promising alternative approach to the treatment of several chemotherapy resistant malignancies.

In its most primitive form, successful eradication of disease was demonstrated with infusion of transplant donor derived unselected lymphocytes in CML patients with relapsed disease after bone marrow transplant [11].Since then, this approach has been further exploited to efficiently generate cytotoxic T-cells directed against specific tumor or viral antigens for eradication of cancer and infections respectively. Substantial efforts from several groups led to the development of techniques for *in-vitro* stimulation and expansion of antigen-specific cytotoxic T-cells, either derived from the patient or volunteer donors. Initial studies, In infusion of *in-vitro* expanded autologous tumor infiltrating lymphocytes (TILs) induced regressions of disease in patients with melanoma, renal cell carcinoma and other tumors [12]. Subsequent studies demonstrated successful *in-vitro* expansion of T-cells responsive to specific peptide determinants of tumor or viral antigens using APCs loaded with peptides or cell lysates. Adoptive transfer of T-cells sensitized against specific TAA such as gp100 and MART-1 and NY-ESO-1 demonstrated clinically significant responses in the treatment of melanoma and synovial sarcoma in selected patients [13–16].

Despite its clinical successes, T-cell therapy has had its limitations in the availability and generation of therapeutic T-cells for a larger group of patients. *in-vitro* expansion of each of these types of T-cells on a clinical scale providing adequate doses for effective treatment requires the use of specific conditions and cytokines permitting such expansion. Approaches aimed at reproducibly achieving such large scale expansions have been developed in recent years. This review will focus on cell based artificial antigen presenting systems (AAPC).

## Fundamentals of T-cell Activation: The T-cell – APC Interaction and Co-Stimulation

T-cells require several signals to become activated and perform their function. The first signal imparted is when the T-cell receptor interacts with the corresponding MHC on an APC. The next required signal is that of co-stimulation, provided upon binding of the

**Figure 2:**
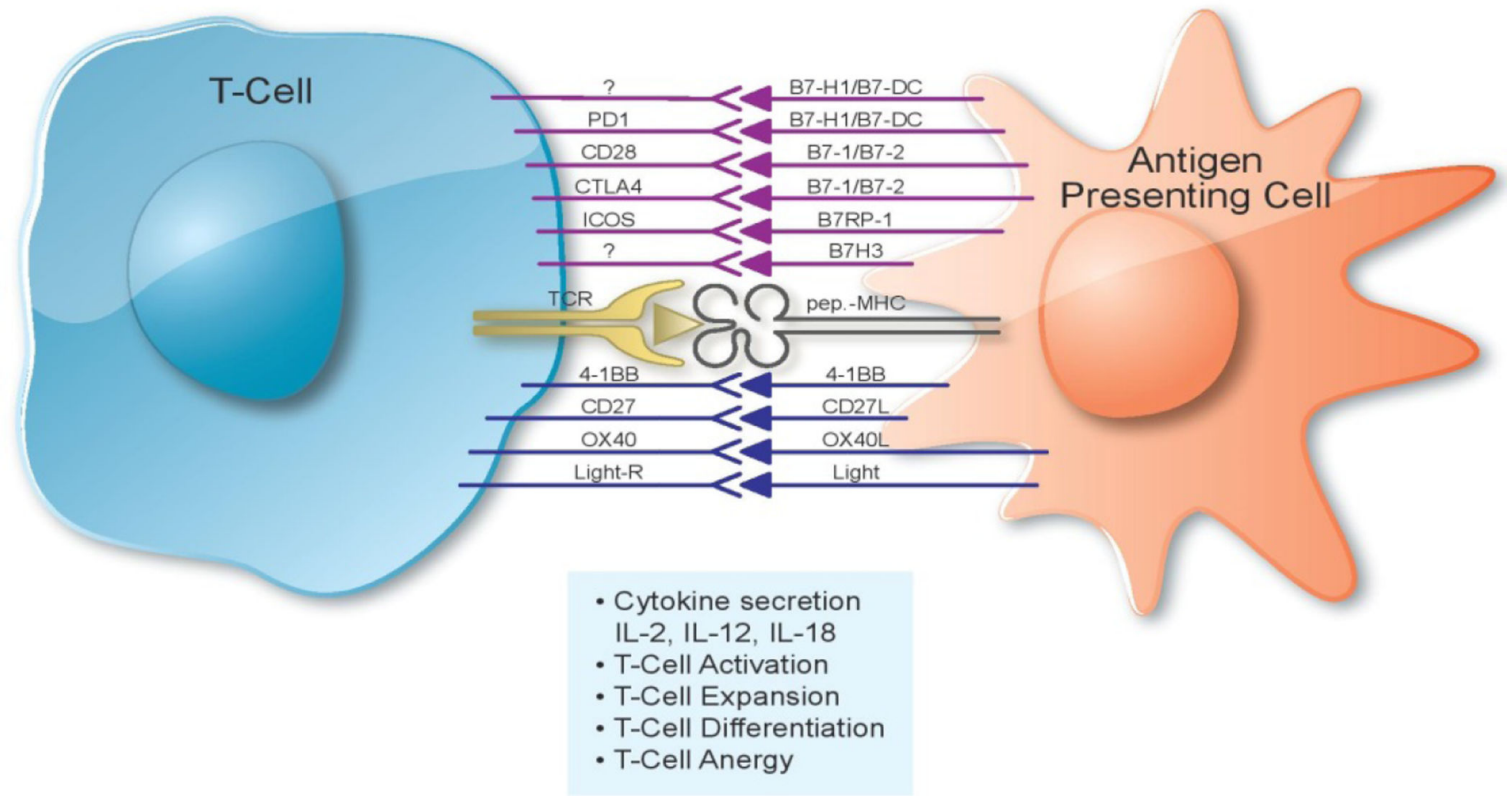
The T-cell APC Interface. T-cells receive sequential signals to become functionally active. The engagement of the T-cell receptor with the targeT-cell expressing the appropriate MHC-peptide complex serves as a priming signal for T-cells. Following this the T-cells require specific signals at the T-cell APC interface to become functionally active and either lyse targeT-cells or serve as regulatory T-cells. The molecules involved in these interactions; either co-stimulatory or inhibitory, are depicted in this figure.

TCR with the MHC-peptide complex, wherein molecules such as CD80 or anti-CD28 expressed on the APCs bind to their ligands expressed on T-cells (Figure 2). The last signal is conferred by cytokines released by the T-cell and the APC that allow for growth and expansion of the desired T-cells. These signals are typically provided by antigen presenting cells such as a dendritic cell (DC).

Dendritic cells (DC) are professional antigen-presenting cells (APC) that have an extraordinary capacity to stimulate naive T-cells and initiate primary immune responses to pathogens. They are continuously generated in the bone marrow and are widely distributed as immature DC to both lymphoid and non-lymphoid tissues [17]. The DC have not been assigned a definitive hematopoietic “lineage” since there are no defining lineage-specific markers (likeTCR rearrangement for T-cells). These cells uniquely arise through “convergent” hematopoiesis, from progenitors at various stages of differentiation ranging from the CD34+ hematopoietic progenitor cells (HPC) [18,19], to terminally differentiated monocytes [20]. The definition of DC therefore relies on a constellation of phenotypic and functional characteristics including morphology, expression of surface markers, cytokine/chemokines and transcription factors (e.g., RelB), and their function. The signaling pathways contributing to DC differentiation include PKC, MAP kinase, NFkB and relb. *in-vitro*, DC have been successfully derived using a combination of cytokines typically including IL-3, SCF, Flt3L, IL-6, GMCSF, TNF-α, IL-4, and IL-lβ.

**Figure 3:**
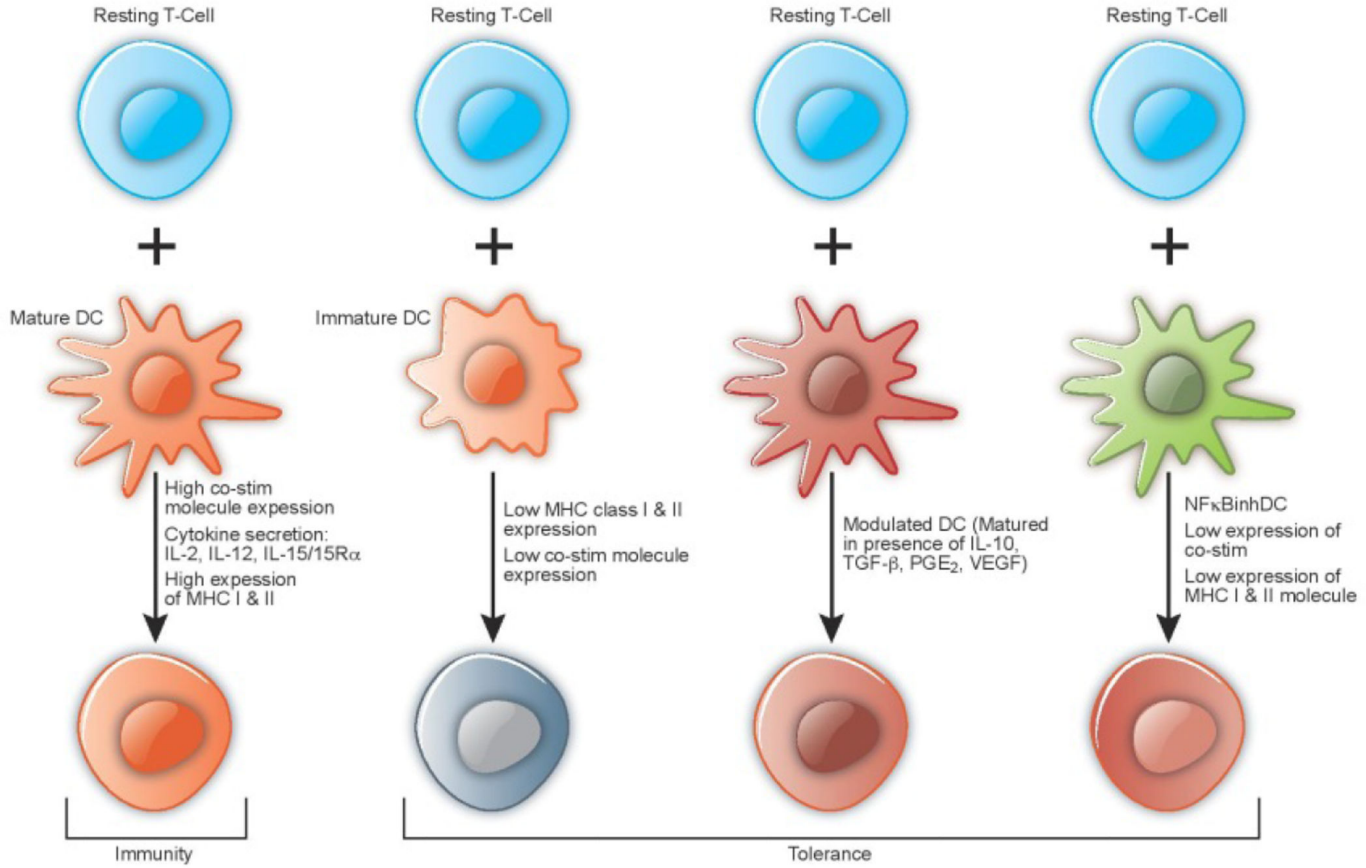
Subtypes of Dendritic Cells Affecting T- cell function. Cytokine signals are critical in the generation and maturation of DCs, which are the professional antigen presenting cells. Mature DCs are endowed with optimal surface expression of MHC as well as T-cell co-stimulatory molecules, and offer an optimal environment to cytotoxic and helper Th1 type T-cell signaling and expansion. DCs that are in a so-called ‘immature’ state, are unable to stimulate T-cells due to the lack the requisite accessory signals for T-cell activation, such as CD40, CD54 and CD86. These DCs play a central role in the development of a T-cell repertoire that is tolerized to self-antigens. This occurs in the thymus (central tolerance) by deletion of developing T-cells, and in lymphoid organs (peripheral tolerance) probably by the induction of anergy or deletion of mature T-cells. DC function can also be modulated in the presence of specific cytokines such as IL-10, TGFβ, and by inhibition of NFkB signaling, again leading to induction of tolerance. Therefore, the DC system that initiates immunity to foreign antigens also appears to tolerize T-cells to self-antigens.

DC can secrete specific cytokines, which endows them with the ability to stimulate Th1, Th2 or Treg subtypes of T-cells upon TCR engagement depending upon the cytokine conditions (Figure 3).

## Limitations of DC for Clinical Application of Adoptive T-cell therapy: Role for Artificial Presenting Cells

The limited availability of cells constitutes a serious obstacle to the use of DC for vaccine therapies or for generating T-cells for adoptive immunotherapy. DC generated for clinical use are derived from the peripheral blood monocytes of patients or transplant donors. This requires a large amount of blood or leukapheresis to be collected, which is both expensive and time-consuming. In tumor bearing patients, additional constraints with this approach are presented due to the effects of chemotherapy leading to a decreased number of DCs in the peripheral blood, as well as the suppressive cytokines released in the tumor mileu which impair the function of the host DC [21]. The differentiation and maturation of DC is inhibited by the soluble immunosuppressive factors secreted by the tumors such as IL-10, TGFβ, PGE2, and VEGF. These immature DC have abnormally low expression of MHC-II and low or undetectable levels of costimulatory molecules, rendering them incapable of processing and presenting antigens, and therefore, unable to induce an effective immune response against the tumor [22]. Certain tumors may further induce the patients’ own APC to express other costimulatory molecules, like B7.H1, that preferentially stimulate regulatory T-cells to suppress immune responses [23].

To overcome these limitations, induced pluripotent stem (iPS) cells have been explored as a source to derive DC (iPSDC). Tseng et al. and Silk et al. demonstrated successful generation of fully human DC derived from human iPS [24,25]. These iPSDC were also shown by Silk et al. to efficiently cross present the TAA, Melan A to naïve CD8+ T-cells when loaded exogenously with recombinant protein *in-vitro*, stimulating a primary Melan A-specific immune response that could be tracked using tetramer technology [24]. Although this approach offers significant promise for tumor immunotherapy using vaccines, it also necessitates the generation of iPSDC potentially for individual patients. For adoptive immunotherapy applications, this again poses a time constraint towards generating antigen specific T-cells.

An alternative and more practicable approach that has been developed as a resource for antigen presentation are cells that are genetically modified to express the desired T-cell co-stimulatory molecules, human HLA alleles and /or cytokines. Such artificial antigen presenting cells (AAPC) are able to provide the requirements for adequate T-cell engagement, co-stimulation, as well as sustained release of cytokines that allow for controlled T-cell expansion [26]. These cells are not subject to the constraints of time and limited availability and can be stored in small aliquots for subsequent use in generating T-cell lines from different donors, thus representing an off the shelf reagent for immunotherapy applications (Table 1). Expression of potent co-stimulatory signals on these AAPC endows this system with higher efficiency lending to increased efficacy of adoptive immunotherapy. Furthermore, AAPC can be engineered to express genes directing release of specific cytokines to facilitate the preferential expansion of desirable T-cell subsets for adoptive transfer; such as long lived memory T-cells.

## Optimal Therapeutic Features of T-cells for Adoptive Immunotherapy

T-cells are broadly classified as naïve or antigen experienced based on their encounter with antigen and differentiation status. Antigen specific T-cells are further classified based on their differentiation status into central memory (TCM), effector memory (TEM), and terminally differentiated effector cells (TE) [27]. Recent emerging data describes a population of T-cells with stem cell like properties (TSCM) that would have the potential for prolonged persistence and further replication *in-vivo* [28–30]. In earlier clinical trials, adoptively transferred anti-tumor T-cells clones, even when infused in large numbers, demonstrated only limited clinical efficacy, which was primarily attributed to the lack of persistence of the T-cells infused [13,14]. Subsequent studies evaluated the potential of different T-cell subsets with respect to *in-vivo* activity and persistence. In both animal models and humans, recent studies have shown that adoptively transferred TCM phenotype T-cells, with high expression of L-selectin (CD62L), CCR7 and CD44 provide durable immunity against infections such as CMV [31–33]. Berger et al. [31] demonstrated that TCM derived T-cells when adoptively transferred into macaques persisted for prolonged periods *in-vivo* and re-acquired the phenotypic markers of TCM cells, and subsequently Wang et. al. [34] showed prolonged engraftment of TCM derived cells in an immunodeficient mouse model. In TCR transgenic mouse models, Restifo et al. have demonstrated that antigen specific naïve and TCM cells are more effective than TE cells in eradicating large established tumors, and paradoxically, differentiated T-cells displaying high functional activity *in-vitro* were less effective in eradicating tumors *in-vivo* [35]. In recent clinical trials, persistence of adoptively transferred T-cells has been correlated with regression of disease [36]. Therefore, such TCM cells are a desirable T- cell population for adoptive immunotherapy because they have the potential to provide durable protection against disease by virtue of their lymphoid homing properties *in-vivo* [27], lending to their prolonged survival after infusion.

## Artificial Antigen Presenting Cells: Potential Applications for Immunotherapies

AAPC are a developing technology for use in adoptive immunotherapy. AAPC use the kinetics known about antigen presentation, but adapt a platform in which an APC provides specific signals delivered using a designed template to stimulate T-cell expansion. The use of these artificial platforms allow for expression of specific molecules on these cells providing a more controlled stimulation of T-cells, therefore permitting the propagation of T-cells with specific phenotype and activity. AAPCs can be derived from cell lines using viral transduction of genes encoding specific co-stimulatory molecules and/or HLA molecules, or from synthetic materials such as polystyrene coated with specific cytokines and/or co-stimulatory molecules [26,37].

The cell lines that have been used for the synthesis of AAPC are derived from insects (drosophila melanogaster), human (K562), mouse (NIH3T3). In 1996, Sun et al. described this approach using MHC-class-I transfected insect cells as antigen-presenting cells [38]. This work was based on the finding by Jackson et al. that Drosophila melanogaster cells could be successfully transfected with MHC genes, which could then be stably integrated into the genome and could be expressed on the surface of insect cells [39]. They also established that these MHC molecules were empty, and could therefore be stabilized by complexing with β2 microglobulin and exogenous peptides.

Thereafter, Schonberger et al. developed an artificial APC using a mouse embryonic cell line engineered to express the H-2Db-restricted CTL epitope of the human Ad5 EIA protein as well as costimulatory molecules B7.1 or ICAM1 [40]. The choice of costimulatory molecules for engineering these cells was based on concurrent emerging data on the molecules constituting this pathway and their functions. Co-stimulation is the second critical signal provided during activation of T-cells after engagement of the TCR with a cognate peptide–MHC complex (Figure 2). The best known co-stimulatory ligands are members of the B7-family, B7-1 (CD80) or B7-2 (CD86), that are expressed on professional antigen-presenting cells (APCs) such as dendritic cells and bind to CD28 molecule expressed on T-cells [41,42]. CD28 amplifies the signal received through TCR engagement thereby lowering the threshold for T-cell activation, while simultaneously enhancing T-cell survival by upregulating anti-apoptotic proteins such as Bcl-xL and c-FLIPshort, to prevent activation induced cell death [43,44]. Signaling through CD28 supports naïve T-cell activation, proliferation and survival [45–47]. As evidenced in CD28 deficient mice, primary CD8+ T-cell responses to pathogens are not developed in the absence of this signal, which underscores the obligate requirement of CD28 co-stimulation for T-cell priming and activation [48–52]. Stimulation of T-cells using the AAPC developed by Shonberger et al. further validated the critical role of B7.1 co-stimulation for *in-vitro* T-cell stimulation and expansion as well as the advantage of antigen density on the APC for stimulating robust antigen specific T-cell responses.

The subsequently developed cell based AAPC systems were designed to stimulate either non-specific expansion of T-cells or expansion of epitope specific T-cells responsive to determinants within viral or tumor antigens presented by specific HLA alleles.

## AAPC for Non-Specific Expansion of T-cells

Systems for non-specific expansion of T-cells were initiated using magnetic beads coated with anti-CD3 and anti-CD28 antibodies. Initial studies with this artificial non-cell based system demonstrated preferential long-term expansion of CD4+ T-cells [53], however, this AAPC system did not support the long-term growth of purified CD8+ T- cells [54]. Maus et al. attempted to overcome this limitation by engineering a cell based AAPC using additional co-stimulation. The human erythroleukemia cell line K562 was used, which does not express HLA class-I or class-II, but expresses adhesion molecules ICAM-1 and LFA-3. These K562 cells were engineered to stably express the human low-affinity Fcγ receptor, CD32 (K32), and the co-stimulatory molecule human (h) 4-1BB ligand (K32/4-1BBL). The K32/4-1BBL coated with anti-CD3 and anti-CD28 antibodies were then used as AAPCs [55].

Upon TCR engagement, the activated T-cell is poised to respond to the next stimulatory signal via CD28 −B7.1 co-stimulation, which sets the stage for the delivery of further co-stimulatory signals, which occurs by upregulation of additional receptor- ligand pairs on the T-cell and APCs (Figure 3). These receptor/ligands may be involved in sustaining, diversifying, and/or amplifying the immune response. In particular, members of the TNFR/TNF ligand family, including 4-1BB/4-1BB ligand (4-1BBL), CD27/CD70, and OX40/OX40 ligand (OX40L) appear to be important in enhancing T-cell responses after initial activation [56–59]. 4-1BB is expressed on activated CD4 and CD8 T-cells and is absent on resting T lymphocytes [56,60], and its ligand, 4-1BBL is expressed on activated APC including B cells, macrophages, and dendritic cells [61–63]. Co-stimulation via 4-1BB facilitates responses at lower levels of signaling through the TCR–CD3 complex and CD28, and promotes TH1 differentiation in CD4+ cells [64,65]. 4-1BB signals also increase the duration and magnitude of immune responses, and the size of subsequent immune memory compartments [48,66]. These effects of 4-1BBL stimulation on T-cells appear highly desirable in the context of cancer immunotherapy. Indeed, stimulation of this pathway in mice was shown to eliminate large, established, poorly immunogenic tumors [67–69]. In fact, immune stimulation via 4-1BB was shown to induce tumor eradication when CD80 was ineffective [70]; wherein 4-1BBL could synergize with either CD80 or IL12, or an antigenic peptide to effect tumor regression [69,71,72].

The K32/4-1BBL AAPC described by Maus et al. induced long-term expansion of human polyclonal CD8+ T-cells. The CD8+ T-cell cultures remained in exponential growth even after a third stimulation eliciting a 410-fold higher increase in the total number of T-cells than that in cultures stimulated with CD3/28 beads. Several modifications have since been made on this platform permitting expansions of specific T-cell populations. Accordingly, the K32 cells have been engineered to express a wide array of costimulatory molecules, including CD40, CD64, CD40L, CD70 [73], CD80, CD83, CD86, CD137L [74], ICOSL, GITRL, CD134L, to facilitate proliferation of specific immune cell types including T and NK cells [75–78] (Table 2). This K-32 system has been developed under cGMP conditions and implemented for clinical use (Table 2).

The K32 cells have also been widely used as a platform for the large scale expansion of CAR modified T-cells. Cooper et al. [79] introduced the truncated CD19 gene in K32 −41-BBL AAPC to foster the preferential expansion of CD19 CAR+ T-cells for clinical use. The K32/4-1BBL AAPC have also been modified to secrete specific cytokines, and IL-21 and IL-15 genes transduced to express membrane bound IL-15 and IL-21 aimed to yield higher overall T-cell expansions as well as preferential expansion of long-lived TCM phenotype CAR CD19 modified T-cells [79–81]. More recent studies have focused on developing a universal AAPC for the expansion of all CAR modified T-cells. In this effort, Cooper et al. developed a K562 based AAPC engineered to express a ScFv antibody directed against human IgG4 based on the hypothesis that this mAb would be able to cross-link to the CAR gene transduced and activate CAR gene modified T- cells for sustained proliferation. Accordingly, K562 cells were transduced to express the scFv of 2D3 (designated CARL). The 2D3-derived scFv on AAPC was evaluated for ability to propagate not just CD19-specific T-cells, but CAR+ T-cells of alternative specificities. AAPC expressing CARL were compared to AAPC expressing truncated TAA for directed expansion of specific CAR modified T-cells such as GD2G4CAR, 19G4CAR. The CARL expressing AAPC demonstrated efficient expansion of CAR modified T-cells bearing ScFv against a variety of tumor antigens, thus offering a resource for clinical grade expansions of CAR modified T-cells bearing any antigenic specificity [82].

## AAPC for Stimulation and Expansion of Antigen Specific CD8+ T-cells

The expansion and enrichment of antigen specific T-cells from a starting population of polyclonal CD3+ T-cells containing minimal concentrations of the desired T-cells has remained challenging. Two main cell based AAPC systems have thus far been developed and evaluated for this purpose, and for potential application for adoptive immunotherapy. Latouche et al. first described the generation of mouse fibroblast NIH 3T3 cell based AAPC transduced to express a single human MHC class-I allele (HLA A0201) and critical T-cell co-stimulatory molecules as a platform for *in-vitro* expansion of epitope specific T-cells restricted by a single HLA allele [83]. In engineering these AAPC, the choice of co-stimulatory molecules was further improvised in an effort to maximize the effects of signal 1 and 2 for T-cell activation. This AAPC was accordingly transduced to express the co-stimulatory molecule B7.1 and the adhesion molecules LFA-3 and ICAM-1. In addition, these 3T3 AAPC expressing HLA A0201 were also transduced to co-express peptide epitopes of influenza and MART-1 proteins to stimulate the expansion of antigen specific T-cells responding to specific peptide-MHC complexes. Successful generation of epitope specific CD8+ T-cells bearing an effector memory phenotype was achieved using 3T3 AAPC that were directed against both viral and tumor antigens, and were cytotoxic against tumor cell targets as well as peptide loaded targets *in-vitro*. A higher efficiency of T-cell expansion was attained using 3T3 AAPC compared to autologous peptide loaded DC; AAPC yielding 2 fold higher T-cell expansions with a cytolytic activity that was 1.6 to 4-fold higher. Importantly, T-cells generated using these AAPC did not demonstrate activity against targets lacking HLA A0201 or HLA A0201 expressing targets lacking the appropriate antigen, thus establishing the ability of this AAPC system to foster the generation of HLA restricted epitope specific T-cells.

The 3T3 HLA A0201 AAPC system was further validated for expansion of T-cells against other antigens including CMVpp65 [84] as well as telomerase tumor antigen [85]. These cells were further developed into a panel of AAPC, each expressing a single HLA class-I allele as a platform for the expansion of antigen specific T-cells restricted by a desired HLA allele [86]. This panel of AAPC permitted the generation of antigen specific T-cells responding to specific epitopes of CMVpp65 that were restricted by the HLA allele expressed by the sensitizing AAPC. The use of a grid of peptide pools consisting of a defined set of overlapping pentadecapeptides permitted mapping of epitopes eliciting T-cell responses [87,88]. The epitopes eliciting responses in these studies were all previously reported to be presented by the same HLA alleles in man. These studies established that this panel of AAPC can be used to generate T-cells responding to both immunodominant and subdominant epitopes presented by a variety of HLA class I alleles. The T-cells responding to subdominant epitopes demonstrated adequate functional activity *in-vitro*. The *in-vivo* functional activity of T-cells responding to subdominant epitopes in comparison to the activity of T-cells responding to immunodominant epitopes is currently being explored to determine the clinical applicability of this approach for treatment of a broader group of patients. This panel of AAPC, each expressing HLA 0101, A0201, A2402, A1101, B0702, B0801 and C0401 will cover over 95% of a racially diverse patient population. Such a panel of AAPC, therefore represents an off the shelf resource for the generation of antigen specific T-cells of desired HLA restriction for adoptive immunotherapy of patients of any ethnicity inheriting diverse HLA alleles.

The K-562 cell line is another cell based AAPC system developed for stimulating antigen specific T-cell expansion *in-vitro*. K562 cells were transduced to co-express HLA A0201 as well as the T-cell co-stimulatory molecules CD80 and CD83 [89]. These cells when loaded with different viral or tumor antigens, were shown to support the priming and prolonged *in-vitro* expansion of antigen specific T-cells displaying a central/effector memory phenotype, with specific cytotoxic activity, and that could be maintained in culture for periods of up to 1 year [90]. A concern with the use of K562 cells is that the expression of HLA may be upregulated on these cells in the presence of peptides, thus providing conditions stimulating the generation of alloreactive T-cells that carry the risk of GvHD upon infusion [91]. An additional concern arises due to the expression of human MHC class I chain-related genes MICA and MICB on K562 cells, which, on the one hand, can be a significant alloantigen [92–94], and, on the other, can release soluble MICA and MICB, which can interfere with CD8 + T-cell effector functions by down-regulating T-cell surface expression of NKG2D [95].

## AAPC for Stimulation and Expansion of Antigen Specific CD4+ T-cells

In order to achieve durable T-cell immunity, CD4+ T-cell help is critical. Indeed, in patients receiving adoptively transferred cytomegalovirus specific CD8+ T-cells, the infused CD8+ T-cells were only shown to have long term *in-vivo* persistence in the presence of CMV specific CD4+ T-cells [96]. Yee et al. have subsequently demonstrated complete regression of metastatic melanoma upon infusion of cloned CD4+ T-cells directed against NY-ESO-1, suggesting that CD4+ T-cells can potentially mediate direct effector function in addition to providing help to effector CD8+ T-cells [15]. Therefore, approaches for the generation of CD4+ T-cells are critical to enhance the success of adoptive immunotherapy. Nadler et al. first reported the development of an AAPC system for the generation Th1 type CD4 + T-cells. In this report, the previously described K562 cells expressing CD80 and CD83 [89] were used as the backbone, and were successively transduced to co-express HLA class – II alleles DRB1 0101 and DRB1 0701 as well as CD64, the common Fc γ receptor, the invariant chain (li) and the α and β chain of HLA DM. These cells were then used for the generation of AAPC expressing HLA class-II alleles, DRB1 0101 and DRB1 0701 [97]. These studies demonstrated successful expansion of DR1 and DR7 specific T-cells responding to CMVpp65 as well as MART1, bearing a Th1 cytokine profile in response to specific antigenic stimulation *in-vitro*.

We have developed a panel of NIH3T3 based AAPC expressing a panel of HLA class II alleles: HLA DRB1 0301, 0401, 0701, 1101 and 1501 [98]. Sensitization of T-cells from CMV seropositive donors permitted the generation of HLA class-II restricted CMV specific T-cells of Th1 phenotype that were responsive to CMV pp65 epitopes previously reported to be presented by HLA class-II alleles. Importantly these studies have allowed us to identify novel epitopes presented by HLA class-II alleles, which could serve as a useful resource to map epitopes for development of refined immunotherapy and vaccine approaches.

## Future Directions

Recent studies have led to the identification of T-cell subsets with the capacity for longer *in-vivo* persistence and the cytokines regulating the propagation of such T-cells. This knowledge has launched the development of a new generation of AAPC specifically engineered to deliver cytokine cocktails facilitating the expansion of TCM and TSCM cells for adoptive immunotherapy. Interleukin-15 is a γ chain cytokine that is critical for the survival and homeostatic proliferation of NK cells and memory phenotype CD8 T-cells [99–101]; and in the presence of antigen, it specifically induces the proliferation of TCM phenotype antigen specific CD8+ T-cells [102–104]. IL-15 mediates its functional activity by binding with its unique high affinity receptor subunit IL-15Rα forming an IL-15Rα /IL-15 complex (15Rα/15) which then shuttles to the cell surface to bind with the β (CD122) and common γ chain subunits to initiate signaling in receptive lymphocytes [105–107]. We generated HLA A0201+ NIH 3T3 based AAPC that were also transduced to co-express IL15Rα and IL-15 genes. T-cell stimulation using IL15Rα/IL-15 expressing AAPC fostered the preferential expansion of antigen specific T-cells bearing a TCM phenotype [108]. We and others have shown that IL-15 can prolong the *in-vivo* persistence of antigen specific T-cells [34], specifically when administered in complex with its high affinity receptor IL-15Ra/IL-15 [109]. AAPC systems expressing and secreting such IL-15Ra/IL-15 complexes may be a useful technique for the efficient generation of TCM cells for clinical applications. IL-21 is another such cytokine that can be developed within this approach. More efficient systems for the consistent expansion of TH1 type CD4+ T-cells need to be developed for clinical use and to further study: (1) for defining epitopes of tumor and viral antigens presented by class-II alleles that would enhance the effect of CD8+ T-cells (2) the functional activity and CD8+ cell help afforded by co-infusion of CD4 and CD8 T-cells *in-vivo* (Table 2).

## Figures and Tables

**Table 1:** Characteristics of artificial antigen presenting cells and professional APCs.

AAPCs	APCs

Generation time consuming, once engineered, can be used or frozen for later use	Generation labor intensive requiring 12 days to several weeks to generate

No variability	Liable to variability

QC issue with each regeneration	QA/QC can be performed on large lots of stored cells

Can select either dominant or subdominant T-cells based on desired HLA restriction	T-cell response to antigen presented is subject to *in-vivo* immunodominance

Can be engineered to deliver specific co-stimulatory signals and cytokines directing expansion of specific cell lineages.	Expess specific set of co-stimulatory molecules and release specific cytokines based on maturation
IL-15 For memory NK and memory T-cell expansion	
IL-21 For priming of naive cells and expansion of memory T-cells	

**Table 2:** Summary of artificial antigen presenting systems and applications.

Clinical					
Artificial APC Backbone	Co-stimulatory Molecules	Cytokine secretion	Antigen- specific/non- specific	TargeT-cell for Expansion	Reference
K562 A2	HLA class I CD80, CD83	nil	MART1	CD3 (CD4, CD8)	Butler et al. [90]
K562	CD64, 4-1BBL	nil	Non specific expansion	CD3 (CD4, CD8)	Suhoski, et al, Mol Ther [75]
K562	CD32, CD80 CD83, CD86 4-1BBL	nil	EBV specific T-cells expanded (K562 used for Co-stimulation)	CD3 (CD4, CD8)	Butler, and Hirano, 2013
K562	CD64, CD86 4-1BBL Truncated CD19, membrane bound IL-15	IL-15	CD19 CAR modified T-cells	CD4, CD8 NK cells	Kebriaei, P. et al. [81]
K562	4-1BBL membrane bound IL-15	IL-15	Non specific expansion	NK cells	Lapteva, et al. [110]
K562	CD64, CD86, Truncated CD19 membrane bound IL-21	IL-21	CD19 CAR modified T- cells	CD8 and CD4	Singh, et al., [79,80]
K562	CD3/CD28 CD86, CD64	nil	Non-specific	Tregs	Hippen, et al, [111]

Non clinical					
Artificial APC Backbone	Co-stimulatory Molecules	Cytokine secretion	Antigen- specific/non- specific	TargeT-cell for Expansion	Reference
K562	CD86, 4-1BB	nil	Nonspecific	Predominant CD8, Low CD4	Gong, et al. [77]
K562	mOKT3, CD80, CD83	nil	Non-specific	CD3	Butler, et al. [78]
NIH 3T3 fibroblast	HLA A0201 + β2 microglobulin, B7.1, ICAM-1, LFA-3	nil	Antigen specific hTERT, CMVpp65, MART1, flu	CD8 T-cells	Latouche et al. [83], Papanicolaou et al., [84], Dupont et al. [85]
NIH 3T3 fibroblast	HLA A0201, A2402, B0702, B0801, C0401 +β2M, B7.1, ICAM-1, LFA-3	nil	CMVpp65 Specific, HLA class-l restricted T-cells	Predominant CD8	Hasan, et al. [86]
K562	CD80, CD70, 4-1BB	nil	MART-126-35, gp100 and Cyp1B1	CD8	Zeng et al. [73]
K562	CD64, CD86, CD137L memb bound IL-15	nil	TILs (melanoma)	Higher CD8, CD4	Forget, et al. [74]
K562	HLA class-ll (DRB1 0101 and DRB1 0701), Ii, HLA DMα, DMβ, CD32, CD64, CD80, CD83	nil	MART-1 and CMVpp65	CD4	Butler, et al. [97]
NIH 3T3 fibroblast	HLA Class-ll (DRB1 0301, 0401, 0701, 1101, 1501), li B7.1, ICAM-1, LFA-3	nil	CMVpp65	CD4	Hasan, et al. [98]
K562	CD1d, CD80, CD83	nil	α-galactosyl ceramide	iNKT-cells	Imataki, et al. Blood
K562	CD19, CD64, CD86, CD137L, IL-15	nil	OKT3	CAR modified T-cells	Singh, et al., [79]
